# Research progress of exosomes in drug resistance of breast cancer

**DOI:** 10.3389/fbioe.2023.1214648

**Published:** 2024-01-04

**Authors:** Lihui Liu, Daqing Jiang, Shi Bai, Xinfeng Zhang, Yue Kang

**Affiliations:** ^1^ Department of Breast Surgery, Liaoning Cancer Hospital and Institute, Cancer Hospital of Dalian University of Technology, Cancer Hospital of China Medical University, Shenyang, China; ^2^ Liaoning University of Traditional Chinese Medicine, Shenyang, China; ^3^ School of Information Science and Engineering, Shenyang University of Technology, Shenyang, China

**Keywords:** exosomes, breast cancer, drug resistance, review exosomes, review

## Abstract

Since breast cancer is a heterogeneous disease, there are currently a variety of treatment methods available, including chemotherapy, endocrine therapy, molecular targeted therapy, immunotherapy, radiation therapy, etc. Breast cancer recurrence and metastasis, despite many treatment modalities, constitute a considerable threat to patients’ survival time and pose a clinical challenge that is difficult to tackle precisely. Exosomes have a very special and crucial role in the treatment of drug resistance in breast cancer as a carrier of intercellular communication in the tumor microenvironment. Exosomes and breast cancer treatment resistance have been linked in a growing number of clinical investigations in recent years. This paper covers the status of research on exosomes in the treatment of breast cancer drug resistance and offers theoretical guidance for investigating new strategies to treat breast cancer drug resistance.

## 1 Introduction

Breast cancer (BC) accounts for more than half of all cancer-related deaths in women globally and is the most often diagnosed kind of the disease (F et al., 2018). In addition to causing a serious blow to patients’ quality of life and economic burden, BC is also a significant public health risk due to an increased prevalence and incidence of BC mortality among women. The global BC epidemic has been estimated at 1.6 million new cases per year with more than 50% of these being newly diagnosed. BC accounts for approximately 30% of all cancers diagnosed in developed countries and 40% in developing countries, including India, China, Brazil, South Africa, and Mexico (Sung et al., 2021). In the actual clinical setting, the primary therapies for BC consist of endocrine therapy, targeted medication therapy, chemotherapy, surgical resection, and so forth (Jayaraj et al., 2019). Some studies have found that several immunotherapy drugs have shown good efficacy in clinical trials, but they are not widely used in clinical practice (Schmid et al., 2018). Nevertheless, medication resistance and a dearth of biomarkers for monitoring therapeutic response could occasionally make therapies less effective. Therefore, it is essential to understand the potential biological mechanisms underlying pharmaceutical resistance and to hunt for reliable biomarkers to predict and monitor therapy response.

**FIGURE 1:**
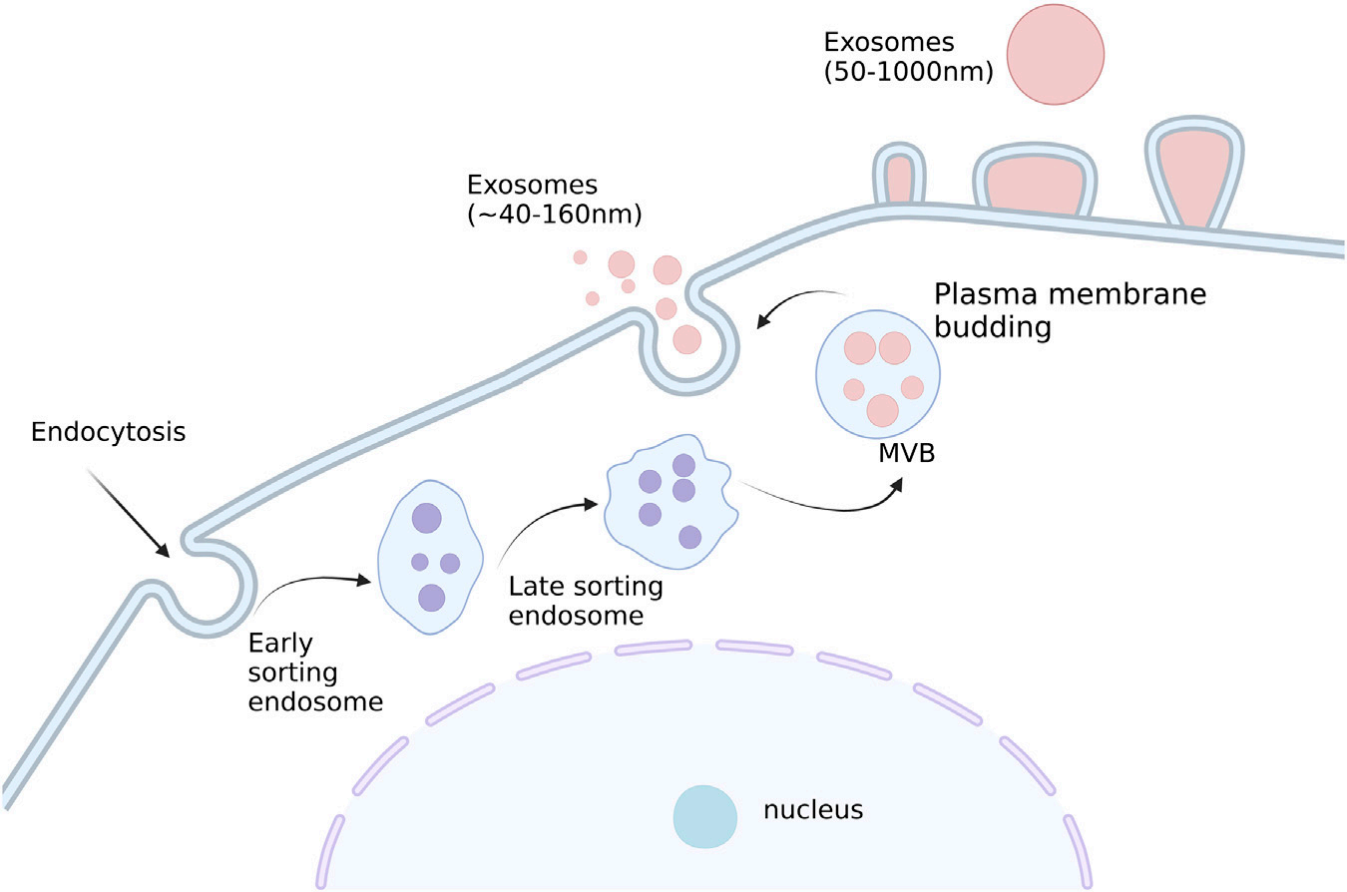
Schematic representation of the exosome production process. Small vesicles are produced by endocytosis, which fuse to form early nuclear endosomes and gradually becomes late nuclear endosomes. With the entry of some ‘cargo ' such as miRNA, enzyme molecules, and heat shock proteins in the cytoplasm, many small vesicles are produced in the late nucleus and gradually evolve into multivesicular bodies. Subsequently, these vesicles will be released extracellularly to form exosomes. Created with BioRender.com.

Extracellular vesicles (EVs) are tiny membrane vesicles that exist outside of the cell formed by cells that transport a range of functional nucleic acids, proteins, and lipid cargos required for intercellular communication. (Sedgwick and D’Souza-Schorey, 2018; Maacha et al., 2019; Sedgwick and D’Souza-Schorey, 2018; Mao and Jin, 2019). The dual invagination of the plasma membrane and the development of intracellular multivesicular bodies (MVBs) that contain intraluminal vesicles (ILVs) result in the production of these exosomes. ILVs are finally discharged as exosomes through exocytosis and MVB fusion to the plasma membrane, with sizes varying from around 40–160 nm. The classification of EVs is in a constant state of flux, but they are generally split into two major collectives: ectosomes and exosomes. The former includes the EVs, which can be found in the plasma membrane; and intracellular vesicles, which may contain proteins that can bind to receptors on cells, such as adhesion molecules or cytokines. The latter include endoplasmic reticulum-bound proteins, including cytochrome oxidase, proteases, enzymes involved in glycolysis and other metabolic reactions, and cell surface markers (Cocucci and Meldolesi, 2015). Ectosomes are vesicles with dimensions ranging from ∼50 nm to 1 μm that are produced when the plasma membrane buddes straight outward, creating microvesicles, microparticles, and large vesicles. Conversely, though, exosomes originate from endosomes and are in a size range of ∼40–160 nm in diameter (100 nm on average) (Kalluri and LeBleu, 2020). Schematic representation of the exosome production process has shown in Figure 1.

Increasing evidence has pointed to a newly identified mechanism that causes medicine resistance called exosome-mediated cell communication (Maacha et al., 2019; Goh et al., 2020). Exosomes directly export drugs, cause inactivation of drugs, and transfer functional proteins and noncoding RNAs, all of which contribute to resistance to BC. However, the role of exosomal drug transport mechanisms in resistance to BC has not yet been elucidated (Giallombardo et al., 2016; Ender et al., 2019). Different sources of exosomes enter recipient cells through endocytosis. This process has three different mechanisms (Kalluri and LeBleu, 2020): 1). The ‘cargo’ of the exosomes is discharged into the cytoplasm and reformed into multivesicular bodies after they enter the recipient cells. 2). Exosomes ‘cargo’ is released into the cytoplasm but fuses in conjunction with the plasma membrane. 3). Exosomes can transport ‘cargo’ into the cell by endocytosis when their ligands attach to certain receptors on the receptor cell membrane.

## 2 Drug resistance status of BC

The capacity of infiltration and migration in BC is one of their traits that influences tumor patient survival and may even result in mortality. Breast tumor cells can spread to different places in the body using a variety of molecular mechanisms and pathways (Rashid et al., 2021; Tan et al., 2021; Wang et al., 2021b). BC metastasis is caused in various ways. For instance, the BC drug has-circ-0068631 engages EIF4A3 and causes c-Myc signaling to increase BC metastasis (Wang et al., 2021a). There are elements that prevent BC migration and invasion. CST6 peptides and protein inhibit CTSB activity to prevent BC from encroaching into bone (Li et al., 2021b). In clinical courses, focusing on variables related to BC metastasis has proven helpful (Hou et al., 2021). It has been demonstrated that extracellular Hsp90α promotes lymph node invasion in BCers and that cancer metastasis can be inhibited by employing the appropriate antibody (Hou et al., 2021). NFE2L3 downregulation (Dai et al., 2021). It has been proposed that overexpressing MTA1 increases BC metastasis. FOXP3 inhibits MTA1 expression to decrease the spread of BC (Liu et al., 2021). Consequently, a number of molecular mechanisms influence the regulation of BC metastasis (Guo et al., 2021; Li et al., 2021a; Yang et al., 2021b; Zuo et al., 2021). Some strategies have been used in impairing BC invasion. For example, element nano-emulsions reduce the spread of breast carcinoma cells by inducing reactive oxygen species (ROS) scavenging (Han et al., 2021). Anti-cancer agents such as alkaloid derivative ION-31a (Ni et al., 2021) and adducing formula (Yang et al., 2021a) can suppress BC metastasis by affecting autophagy and Hsp90α. The mechanisms behind the epithelial-to-mesenchymal transition (EMT) and how they impact the growth of breast tumor cells are the subject of the following sections.

In cancer, up to 90% of deaths are due to drug resistance in humans, and the number is still increasing It has reached a level where there will be no cure for cancer at this moment (Łukasiewicz et al., 2021). Chemotherapeutic medications are less effective when there is multi-drug resistance (MDR), which frequently results in metastasis and relapse. About half of individuals who are resistant to drugs have either acquired or innate resistance (Wang et al., 2019b). The development of new therapies which can overcome these resistance mechanisms is crucial to achieving a cure for cancer. Three factors can cause intrinsic resistance to emerge before therapy does: genetic alterations, the expansion of pre-existing insensitive fractions (such cancer stem cells), and the natural defense against dangerous external substances (Holohan et al., 2013). Alternatively, acquired resistance may result from activation of proto-oncogenes, changes in gene expression due to mutations or epigenetic marks as well as changes in the tumor microenvironment following treatment (Holohan et al., 2013). There are several mechanisms of resistance in BC, which includes increased drug efflux, enhanced DNA repair, senescence escape, epigenetic modifications, tumor heterogeneity, tumor microenvironment (TME), and EMT (Holohan et al., 2013; Cosentino et al., 2021; He et al., 2021). Exosomes play a significant and particular role in the management of BC resistance. More details are as follows.

## 3 Exosome and chemotherapy drug resistance in BC

Chemotherapy is one of the most common treatments for invasive BC, especially triple-negative breast cancer (TNBC). To avoid cell death brought on by chemotherapeutic medicines, BC cells can, nevertheless, use several strategies. These processes mostly involve drug efflux and inactivation (Dallavalle et al., 2020), activation of bypass signaling or pro-survival pathways, enhancement of DNA damage repair (Battista et al., 2020), and induction of EMT (Navas et al., 2020) and stem-like property. In terms of tumors, tumor-derived exosomes (TDEs) are involved in regulating tumor growth, invasion, drug resistance, angiogenesis, immune evasion, and remodeling of the tumor microenvironment (Wan et al., 2020). Furthermore, a great deal of research has shown that the cell stress brought on by anticancer therapy altered the makeup of exosomes secreted by tumor cells. Treatment resistance may arise from drug-resistant phenotypes spreading throughout BC tumor cells (Lv et al., 2014a).

### 3.1 Exosomes-mediated drug efflux and inactivation

**FIGURE 2:**
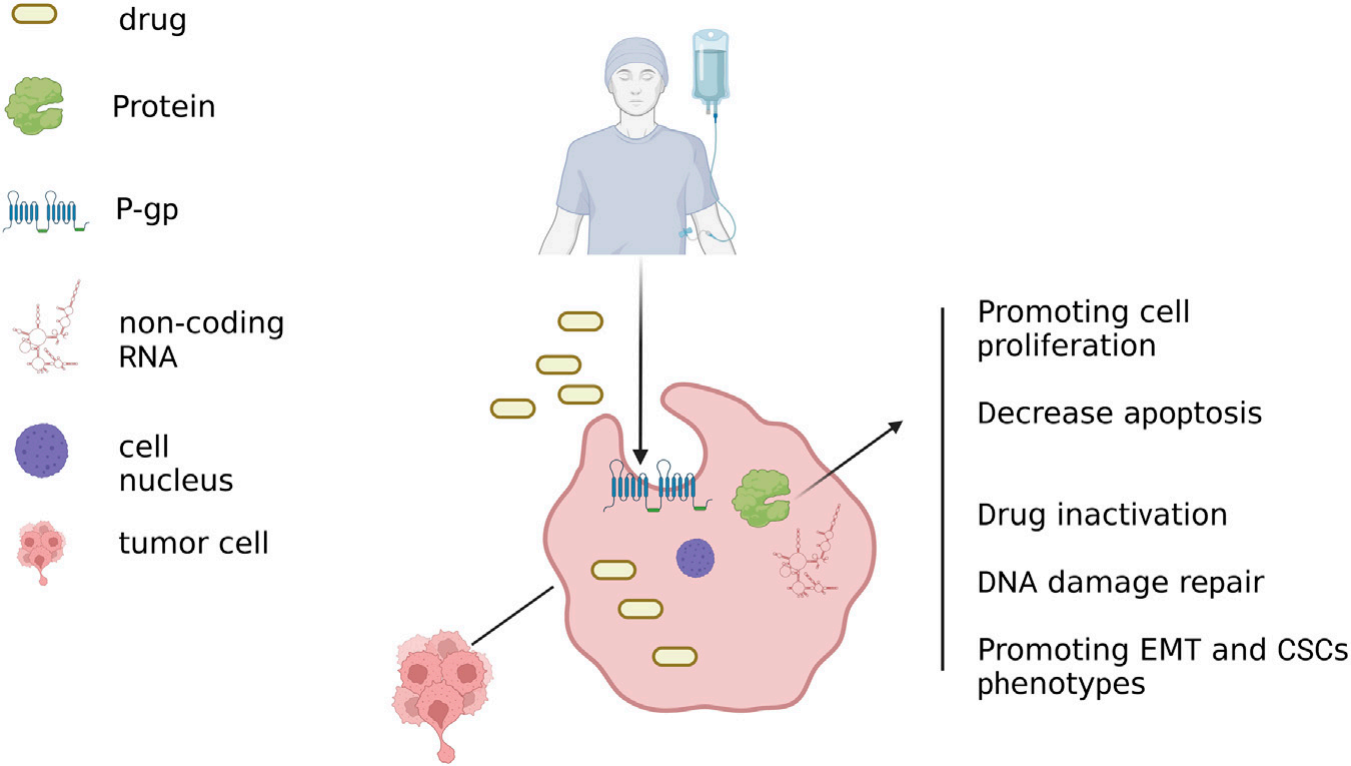
Exosome-mediated pathways of BC chemoresistance. When chemotherapeutic medications are enclosed in exosomes, they secrete. To increase drug efflux, exosomes help membrane-embedded drug efflux pumps spread horizontally to cancer cells that are vulnerable to them. Additionally, exosomes deliver advantageous proteins and miRNAs that boost P-gp expression in cancer cells that are susceptible. Bioactive payloads carried by exosomes promote the growth, survival, drug resistance, repair of DNA damage, EMT, and stem-like characteristics of cancer cells. Created with BioRender.com.

Cytotoxic medications must accumulate sufficiently within cancer cells to be effective in treating cancer. However, increased drug efflux could lead to chemoresistance. In cancer cell lines, EV shedding-related gene and treatment resistance were found to be positively correlated as early as 2003 (Shedden et al., 2003). They discovered that the chemotherapy agent doxorubicin (DOX) could be exported by BC cells into the extracellular media by vesicle formation (Shedden et al., 2003). One pathway for resistance to therapy in cancer cells was the transfer of membrane-enclosed drug efflux pumps via exosomes to susceptible cancer cells. A schematic diagram of the exosome-mediated BC chemotherapy resistance mechanism has been shown in Figure 2. These pumps export a variety of contaminants, especially anticancer medicines with different structures and functions, using ATP, such as the ATP binding cassette transporter (ABC) (Locher, 2016; Nedeljković and Damjanović, 2019). According to some research, P-gp can travel by exosomes from drug-resistant tumor cells to susceptible cells, resulting in the development of drug resistance (Lv et al., 2014b). This transfer resulted in the acquisition of drug resistance *in vitro* and *in vitro* (Levchenko et al., 2005; Bebawy et al., 2009; Sousa et al., 2015). The other approach involved the modification of P-gp expression by the transfer of useful proteins and miRNAs via exosomes. In drug-resistant BC cells, P-gp was upregulated primarily by transient receptor potential channels (TRPCs) (Ma et al., 2012). P-gp and UCH-L1 protein abundance was greater in ADM-resistant MCF-7 exosomes. LDN-57444 reduced medication resistance in sensitive MCF-7 cells caused by exosomes from ADM-resistant MCF-7 cells internalizing, a UCH-L1-specific inhibitor (Ning et al., 2017). These findings imply that the chemoresistance of BC to chemotherapy was greatly influenced by both the direct export of chemotherapeutic medicines as well as the control of the transfer of or modulation of the drug efflux pump via exosomes. However, exosomes also could deliver the enzymes necessary for drug metabolization, which leads to drug inactivation. Yang et al. (Yang et al., 2017) found that the exosomes of ADM-resistant cells had significantly greater GSTP1 mRNA expression. Exosome-exposed sensitive cells displayed a phenotypic that was resistant to drugs.

Exosomes have also demonstrated outstanding effectiveness in combating medication resistance in various malignant cancers. Some researchers, for example, have created a multifunctional nanoplatform based on hybrid-shelled hydroxychloroquine-loaded hollow ZnS spheres for photodynamic therapy/chemotherapy of glioblastoma (Liu et al., 2023b). Additionally, it has been discovered that DARS-AS1 siRNA can be delivered via EXOs-CL4 and utilized as a novel treatment approach for DOX-resistant TNBC. Meanwhile, EXOs-CL4 can be used as an effective drug delivery system for targeted TNBC treatment (Liu et al., 2023b).

### 3.2 Exosomes mediate the transport of bioactive substances

Exosomes carry bioactive cargo and stimulate unchecked cell cycle progression and pro-survival signaling, which is a feature that aids in the growth and development of malignant tumors. For example, exosomes from several cancer types contain the pro-survival protein (Khan et al., 2011; Ender et al., 2019). According to Kreger et al.'s findings, paclitaxel (PTX) therapy caused MDA-MB-231 cells to secrete survivor-enriched exosomes, which greatly aided fibroblast and SK-BR-3 cell survival after being served with PTX (Kreger et al., 2016). In an exosomal miR-423-5p dependent way, exosomes derived from cisplatin-resistant TNBC cells (231/DDP) modified the susceptibility of other BC cells to DDP (Wang et al., 2019a). By increasing cell proliferation, metastasis, and anti-apoptotic signaling, they were able to impart cisplatin-resistant phenotypes to recipient cells. According to Wang et al. (Wang et al., 2020), the abundance of exosomal long non-coding RNA (lncRNA)-H19 caused BC cells to develop DOX resistance. The resistance to DOX was considerably lowered when lncRNAH19 was inhibited. This rule demonstrated proinflammatory cytokine-suppresses rent for functional proteins, such as including non-coding RNA was one of mechanism underlying chemoresistance.

Numerous large-scale studies have identified the expression patterns of exosomal proteins and miRNAs in BC after chemotherapy (Zhong et al., 2016; Kavanagh et al., 2017; Chen et al., 2018b; Chen et al., 2019; Ozawa et al., 2018). It has been determined that the proteins caveolin-1 (CAV1) and enascin C (TNC) found in EVs produced from BC cells promote the development of BC (Campos et al., 2023). These findings imply that tumor-derived exosomes could significantly contribute to chemotherapy resistance in BC.

## 4 Exosome and hormonal resistance in BC

**FIGURE 3:**
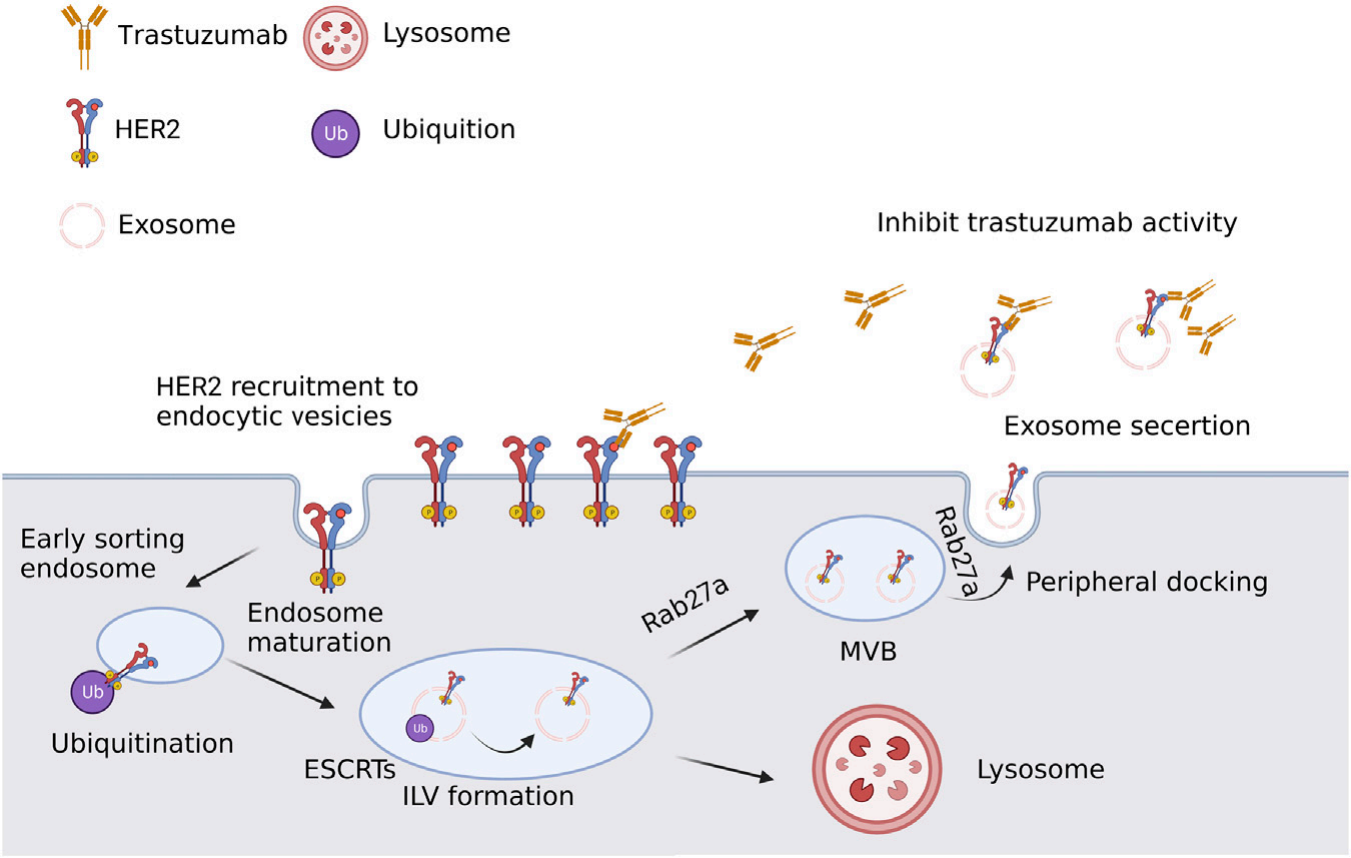
A simple schematic description of the resistance mechanism of HER2 targeted therapy. In developing endocytic vesicles that join with early endosomes, plasma membrane components are grouped. Sorted early endosome materials develop into intraluminal vesicles (ILV)-containing multivesicular bodies (MVBs). The endosomal sorting complex needed for transport (ESCRT) proteins cause ubiquitinated HER2 to collect into patches in the membrane during ILV formation. The majority of MVBs release exosomes to the extracellular space through lysosomal fusion, which destroys their cargo. Created with BioRender.com.

Targeting the estrogen receptor (ER), which is present in large quantities in roughly 70% of BC patients, is an available hormone therapy (Muluhngwi and Klinge, 2017; Brufsky and Dickler, 2018). Endocrine therapy has increased the number of ER-positive BC patients who survive without developing a disease, however, the clinical problem of BC metastasis or recurrence brought on by endocrine resistance has not yet been resolved. There have been several research on the mechanisms of endocrine medication resistance, mostly concentrating on somatic cell alterations, epigenetics, and tumor microenvironment, but the precise mechanisms are still not completely understood. The mechanism of endocrine resistance is generally understood to be quite complex. Many ER-blocking medications are currently on the market and are often utilized in the therapeutic treatment of individuals with ER + BC. One such drug is tamoxifen (TAM), which can successfully shut down ER activation and downregulate the growth of ER + tumors (Early Breast Cancer Trialists’ Collaborative Group EBCTCG, 2011). However, the growth of cancers that gain hormonal resistance after prolonged treatment frequently renders hormone therapy in BC ineffective in BC (Osborne and Schiff, 2011; Rani et al., 2019). The mechanism of hormone resistance is the subject of extensive research. The resistance to hormones primarily arises from dysregulation of estrogen receptors, the activation of several pathways and an imbalance between activators and inhibitors (Osborne and Schiff, 2011; Scherbakov et al., 2012; Muluhngwi and Klinge, 2017; Semina et al., 2018). The ER plays a crucial role in regulating several physiological processes, such as immune system responses (including autoimmune response), metabolism, reproduction, cell proliferation, and many other functions. In short, exosomes could transfer the acquired hormone resistance of BC cells primarily through the following mechanisms: activation of hormone-independent pathways and ER dysregulation caused by exosomal miRNA and protein. Only a handful of studies have demonstrated the transfer of hormone resistance between BC cells. Therefore, more investigation is needed to examine the proteome and non-coding RNA profiles of exosomes released by hormone-resistant BC, as well as to identify the critical elements for the exosome-mediated transmission of the hormone-resistant phenotype. Treatment resistance and exosomes in British Columbia for human epidermal growth factor receptor 2 (HER2) overexpression of HER2 was associated with a poor prognosis for BC (Cortesi et al., 2015). When used in clinical practice, HER2-targeted treatment effectively treats HER2+ BC (Tagliabue et al., 2010). The first monoclonal HER-2 antibody approved for the treatment of HER2+ BC is trastuzumab (Cameron et al., 2017), which dramatically prolongs patients’ lives. Trastuzumab is safe and effective in multiple trials with good safety profiles. However, there are several potential adverse events associated with trastuzumab, such as severe nausea, vomiting, rash, and diarrhea. These side effects may not occur when used together with other anti-HER2 drugs or therapies that affect the immune system. Within a year of finishing treatment, the majority of patients become resistant to HER2-targeted medications, despite the fact that BC patients initially respond well to these treatments (Ahmad, 2019). Figure 3 depicts an illustration of the membrane transport pathway involved in the creation and release of multivesicular endosomes, as well as the resistance mechanism of HER2 targeted therapy.

According to certain research, Antibody-based drugs are neutralized by exosomes, which results in BC trastuzumab resistance (Dong et al., 2020). HER2-targeted medication resistance is tightly correlated with levels of programmed death ligand 1 (PD-L1) and transforming growth factor β1 (TGF-1) (Devan et al., 2022). According to Martinez et al. (Martinez et al., 2017), they discovered that these chemicals are transferred by EVs to cause drug-sensitive cells to exhibit the traits of their source cells. Trastuzumab resistance could also be a result of non-coding RNA dysregulation. RNA-binding protein heterogeneous nuclear ribonucleoprotein A2B1 (hnRNPA2B1) was discovered to be crucial for lncRNA AGAP2-AS1 loading into exosomes (Alarcón et al., 2015). Additionally, by examining the Gene Expression Omnibus database’s publicly accessible BC miRNA expression profiling data, Han et al. (Han et al., 2020) discovered that trastuzumab resistance resulted in a downregulation of miR-567 expression. They then found that trastuzumab resistance was caused by miR-567 suppression, but exosomal miR-567 reversed trastuzumab resistance by inhibiting autophagy-related (Lappano et al., 2020). Therefore, these observations highlight the special role that exosomes play in promoting resistance to targeted therapy, either through direct interactions between HER-2 overexpressed exosomes and targeted agents, or through exosome-mediated transcription changes that promotes cell survival through the HER2-independent pathway.

## 5 Exosome and immunotherapeutic resistance in BC

A new chapter in the treatment of cancer has begun with the recent success of innovative anti-cancer immunotherapies (Zhang and Zhang, 2020). Previously, it was thought that bladder cancer, melanoma, and lung cancer were more immunogenic than BCs (Sugie, 2018). According to recent research, TNBC tumors are more immunogenic than other BC subtypes, with higher levels of lymphocyte filtrating and PD-L1 expression (Barroso-Sousa et al., 2020). Which significantly lengthens patients’ lives. Multiple trials with positive safety profiles have demonstrated the efficacy and safety of trastuzumab. However, Trastuzumab has several possible side effects, including severe nausea, vomiting, rash, and diarrhea. When combined with other anti-HER2 medications or immune system-affecting therapy, these side effects might not manifest. (Michel et al., 2020). On 8 March 2019, the FDA granted accelerated clearance for the anti-PD-L1 medication atezolizumab plus nab-paclitaxel for unresectable locally advanced or metastatic TNBC with PD-L1 expression, based on the findings of the IMpassion 130 trial. However, there is still much work to be done in order to achieve the best effect of BC immunotherapy. In addition, it is urgent to discover and apply new biomarkers to predict the response to immunotherapy. Exosomes are crucial for changing the tumor immunological microenvironment. According to reports, tumor cells could have PD-L1, and exosomal PD-L1 prevents T-cell activation, which could help cancer cells avoid antitumor immunity (Chen et al., 2018a). Furthermore, it appears that anti-PD-L1 antibodies cannot completely inhibit exosomal PD-L1. In Poggio et al. study, has shown that the exosome PD-L1 was expressed in cells of human lung epithelium and mediates cell migration through a mechanism similar to the mechanisms of the proteasome, which might lead to increased expression of PD-L1 (Poggio et al., 2019). However, PD-L1 expression varies and changes over time in various BCs. It is known that among the many BC subtypes, basal-like BC cells express the greatest PD-L (Soliman et al., 2014). According to Moneypenny et al. (Monypenny et al., 2018), in BC cells, the endosomal sorting complex required for the transport-related protein ALIX controls the activation of the PD-L1’s surface expression and the epidermal growth factor receptor. This finding suggested that MSCs were a promising model for studying the mechanisms underlying BC and its complications. A recent study has shown that the expression of CD56 on B cells is down. They found that PD-L1, which confered a more immunosuppressive characteristic on BC cells, was more prevalent on the surface of ALIX-depleted cells. Additionally, Wen et al.'s research (Wen et al., 2016) has shown which exosomes are obtained directly from BC cells that have spread far reduced NK activity and T cell proliferation, possibly limiting the anticancer immune response in pre-metastatic organs. Additionally, another study has shown that TGF--mediated inhibition of T cell proliferation by exosomes derived from BC cells (RONG et al., 2016). However, the development of cancer and the resistance to immunotherapy are both significantly influenced by tumor-associated macrophages. Exosomes produced by mesenchymal stem cells (MSC) were found by Biswas et al. (Biswas et al., 2019)to hasten the course of BC. As a result, type 2 macrophages polarize myeloid-derived monocyte-suppressive cells into highly immunosuppressive macrophages near the tumor bed. This finding suggested that MSCs were a promising model for studying the mechanisms underlying BC and its complications.

These findings have significant ramifications for our comprehension of the fundamental mechanisms driving immunosuppression in BC’s TME. To sum up, exosomes transport immunosuppressive chemicals that have been widely investigated in various cancers and are known to impact immune cell activities in a variety of ways. Since the majority of immunotherapy research for BC is still being done at the time of the clinical trial, exosome-mediated immunosuppression is currently being examined, and it needs more research. Exosomes could be used as a prognostic biomarker that may eventually be employed as a non-invasive method to track the effectiveness of immunotherapy in malignancies. Recent studies have shown that EV release characteristics were generally associated with cellular phenotypic modification, such as EMT. Exosomes regulate EMT, cancer stem cell (CSC), and TME in the drug resistance of BC (Fujiwara et al., 2018b; Fujiwara et al., 2018a) and CSC (Eguchi et al., 2018; Hu et al., 2019). EMT and stemness encourage cells to release EVs, and tumor-derived EVs mayactivate EMT and stemness in tumor cells (Eguchi et al., 2020). As a result, the EMT and CSC characteristics are anticipated to favor both exosome-mediated tumor progression and the development of treatment resistance.

Numerous research over the past few decades have shown that the TME had a significant role in determining both treatment resistance and tumor growth, progression, and metastasis (Brown and Giaccia, 1998; Matei et al., 2017; Taube et al., 2018; Vasan et al., 2019; Lappano et al., 2020). EMT is a biological process in which epithelial features are lost and a mesenchymal phenotype is acquired (Bill and Christofori, 2015). During the course of EMT, some biochemical changes occur in cells, including loss of strong cell-cell adhesion and development of invasive, migratory, and antiapoptotic properties. The major effector molecules are the transcription factors that regulate gene expression (i.e., genes involved in apoptosis or DNA damage). Exosomes are a crucial part of the EMT process that results in a more aggressive phenotype for cancer cells. A growing body of research suggests that exosomes may be capable of delivering pro-EMT factors to recipient cells, promoting the development of BC, chemoresistance, invasion, metastasis, and anti-apoptosis, among others (Qin et al., 2016; Donnarumma et al., 2017; Santos et al., 2018; BIGAGLI et al., 2019). According to Liu et al. (Liu et al., 2015), the miR-155 is an essential EMT regulator and CSCs. Santos et al. (Santos et al., 2018) has reported that the upregulation of EMT was linked to miR-155 in DOX- and PTX-resistant cells. In addition to these effects, transfected miR-155 cells also drive EMT. Some authors reported that miR-155 expression was increased in the presence of DOX and PTX, suggesting a direct role for miR-155 in tumor progression.

**FIGURE 4:**
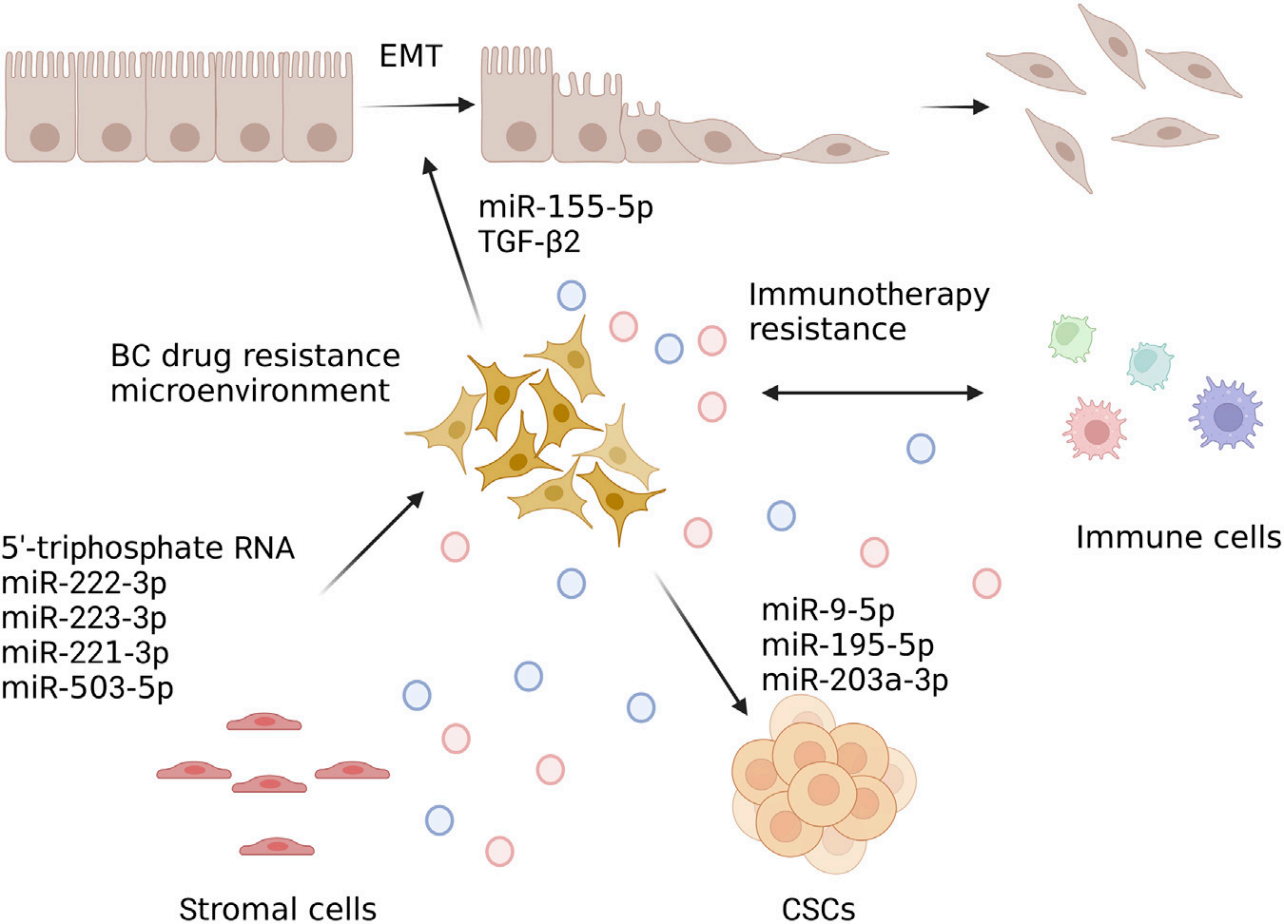
Drug resistance is directionally transferred between TME and BC cells through exosomes. In developing endocytic vesicles that join with early endosomes, plasma membrane components are grouped. Sorted early endosome materials develop into intraluminal vesicles (ILV)-containing multivesicular bodies (MVBs). The endosomal sorting complex needed for transport (ESCRT) proteins cause ubiquitinated HER2 to collect into patches in the membrane during ILV formation. The majority of MVBs release exosomes to the extracellular space through lysosomal fusion, which destroys their cargo. Created with BioRender.com.

Furthermore, immune cells, including myeloid-derived suppressor cells, mast cells, neutrophils, lymphocytes, and macrophages, DCs, and NK cells have been demonstrated to be permeable in the TME (Whiteside, 2016). Exosomes, which carry tumor-associated antigens, interfere with anti-tumor immunotherapy. Exosomes are produced by these cells to transmit information regarding immunosuppression or activation. On the basis of the research reported above, exosomes could be produced by TME cells in response to anticancer therapies and promote TME-to-BC cell interaction that leads to the transmission of drug resistance. Exosomes could be as tumor biomarkers that reflect TME alterations and predict therapeutic response, according to all of these studies. Drug resistance directionally transferred between TME and BC cells through exosomes has shown in Figure 4. The researchers believed that the use of exosomes could help identify new targets for treatment and develop new cancer therapies.

## 6 The potential use of exosomes in treating BC medication resistance

### 6.1 Exosomes as indicators for treatment response prediction

In BC drug resistance management, exosomes could play an important role, for example, using exosomes as markers to predict BC response to treatment. Exosomes are small organelles which have been shown to play an important role in cellular processes including cell growth, differentiation, and survival. It is necessary to make it more routine to dynamically evaluate specific molecular markers and to monitor treatment response and progression with blood-based liquid biopsy analysis (Braden et al., 2014). Dynamic evaluation of particular molecular markers, and blood-based liquid biopsy analysis to monitor treatment response and progression becomes more routine (Bardelli and Pantel, 2017; Campos-Carrillo et al., 2019). However, current methods are not sensitive enough to assess clinical outcomes in patients with solid tumors treated with immunotherapy drugs or other therapies.

Due to its unique characteristics, studies on exosomes in early disease development and as a potential indicator of therapeutic response or resistance are limited. For example, they have relatively stable structures, are found in nearly all biological fluids, carry facial markers, transport payloads that accurately depict the physiological condition of the original cell, and have these characteristics (O’Neill et al., 2019; Nazri et al., 2020; Vanhie et al., 2020). These elements make it easy to understand why a protein or molecule is expressed in a cell only occasionally and not in other areas of the cell, such as when the cell undergoes DNA sequence changes, when the cell suffers damage (such as cancer), or when the cell undergoes during stressful times (such as pregnancy). Exosomes do have certain benefits over other liquid biopsy analysis techniques. Exosomes protect their cargo from spoilage and contain chemicals from their parent cells.

Exosome proteins are not present on the surface of cells, making exosomes a better source for biomarkers than cell surfaces. Thus, in contrast to circulating tumor DNA (ctDNA) or vesicle-free circulating tumor RNA, exosome nucleic acid analysis may be more informative and repeatable. Secondly, exosomes offer the chance to gather information at the DNA, RNA, and protein levels and they are more prevalent than cell-free DNA. Exosome and ctDNA data combined may provide more accurate results or data for tracking the course of BC and forecasting response to BC therapy. The combination of exosomes with ctDNA was not currently available. However, the current study suggested that exosomal protein levels ware significantly elevated in patients receiving BC therapy. In addition, the studies which comparing exosome concentrations in different populations remain largely unexplored.

Exosome-related biomarkers have been studied in several recent investigations in chemotherapy patients. According to Wang et al. (Wang et al., 2017), the amount of TRPC5 expressed in BC tissues and the effectiveness of chemotherapy were substantially connected with the levels of circulating exosomes carrying TRPC5. In addition, higher levels of circulating exosomes expressing TRPC5 after chemotherapy indicated the development of acquired chemotherapy resistance and cancer progression. Therefore, real-time monitoring of chemotherapy resistance can be done by looking for TRPC5-positive exosomes. Another study discovered that cancer patients’ serum levels of the lncRNA HOTAIR were much greater than those of healthy people (Tang et al., 2019). Notably, all patients experienced a significant drop in exosomal lncRNA HOTAIR 3 months following surgery, indicating that the source of serum HOTAIR is tumor tissue and that its level is correlated with the degree of disease invasiveness and tumor burden. It has been shown that the expression of long noncoding RNAs influences cell motility, proliferation, apoptosis, and differentiation. The present study suggested that the expression of lncRNA HotaIR could serve as an indicator for tumor progression and metastasis. Furthermore, a poorer response to neoadjuvant chemotherapy and treatment with TAMs was linked to a high level of serum exosomal HOTAIR expression (Tang et al., 2019). In HER2+ BC and TNBC exosomes, several miRNAs were selectively enriched, as demonstrated by Stevic et al. (Stevic et al., 2018).

TNBC is recognized to be particularly poorly treated due to the lack of specific targeted therapies. However, some researchers (Chen et al., 2017) have used the film method to fabricate curcumin-loaded POCA4C6 micelles (CPM), which are monolayer structure with an average particle size of 3.86 nm. Based on liquid chromatography-tandem mass spectrometry, the micelles exhibited great curcumin encapsulation efficiency and loading. Additionally, *in vitro* investigations revealed that POCA4C6 and curcumin work together synergistically to kill CD44 + CD133 + breast cancer stem cells (BCSCs), and CPM could reduce the self-renewal and aggressiveness of TNBC. These studies not only highlighted the potential of CPM as an effective treatment for TNBC but also demonstrated the novelty and effectiveness of novel nanomaterials in changing drug delivery and anticancer methods, providing us with new ideas for the treatment of cancer stem cells. Drug delivery targeting BCSCs has made extensive use of a range of nanocarriers in recent years, including liposomes, inorganic and polymeric nanoparticles, micelles, and nano-gels. These delivery systems successfully increased medication stability and allowed for the carefully timed delivery of large amounts of multicomponent cargo to BCSCs and/or breast carcinoma cells (He et al., 2016).

Large-scale exosomal biomarker validation studies could provide important information for tumor treatment monitoring. There are still certain restrictions, for instance, there is not any consistent procedure for gathering, processing, and separating exosome samples. Ultracentrifugation is one of the current separation methods, although it takes a lot of time and cannot separate highly pure materials. Based on size separation, immunoaffinity trapping, and exosome precipitation, some alternative techniques have been devised. Due to overlapping properties, these techniques often produce complex mixtures of EVs and other extracellular space components and fail to extract high-purity exosomes (Doyle and Wang, 2019). Additionally, certain microfluidic techniques, such nanofabricated exosome technology, which relies on surface plasmon resonance for label-free detection of exosomes, are not routinely used (Garcia-Cordero and Maerkl, 2020). Exosome enrichment was currently being refined, and each step might be tailored for a particular cargo, like protein, DNA, or RNA (Meldolesi, 2018; LeBleu and Kalluri, 2020). Exosomes are difficult to utilize as biomarkers because they are intermingled with exosomes from normal cells in circulation, making it difficult to distinguish between them and conduct a thorough analysis of tumor-derived exosomes. Using a proteome study of 426 human samples, Hoshino et al. (Hoshino et al., 2020) recently discovered and defined tumor-derived EV markers in human tissue and plasma that differ from normal controls.

### 6.2 Exosomes as novel therapeutic interventions in BC drug resistance

**FIGURE 5:**
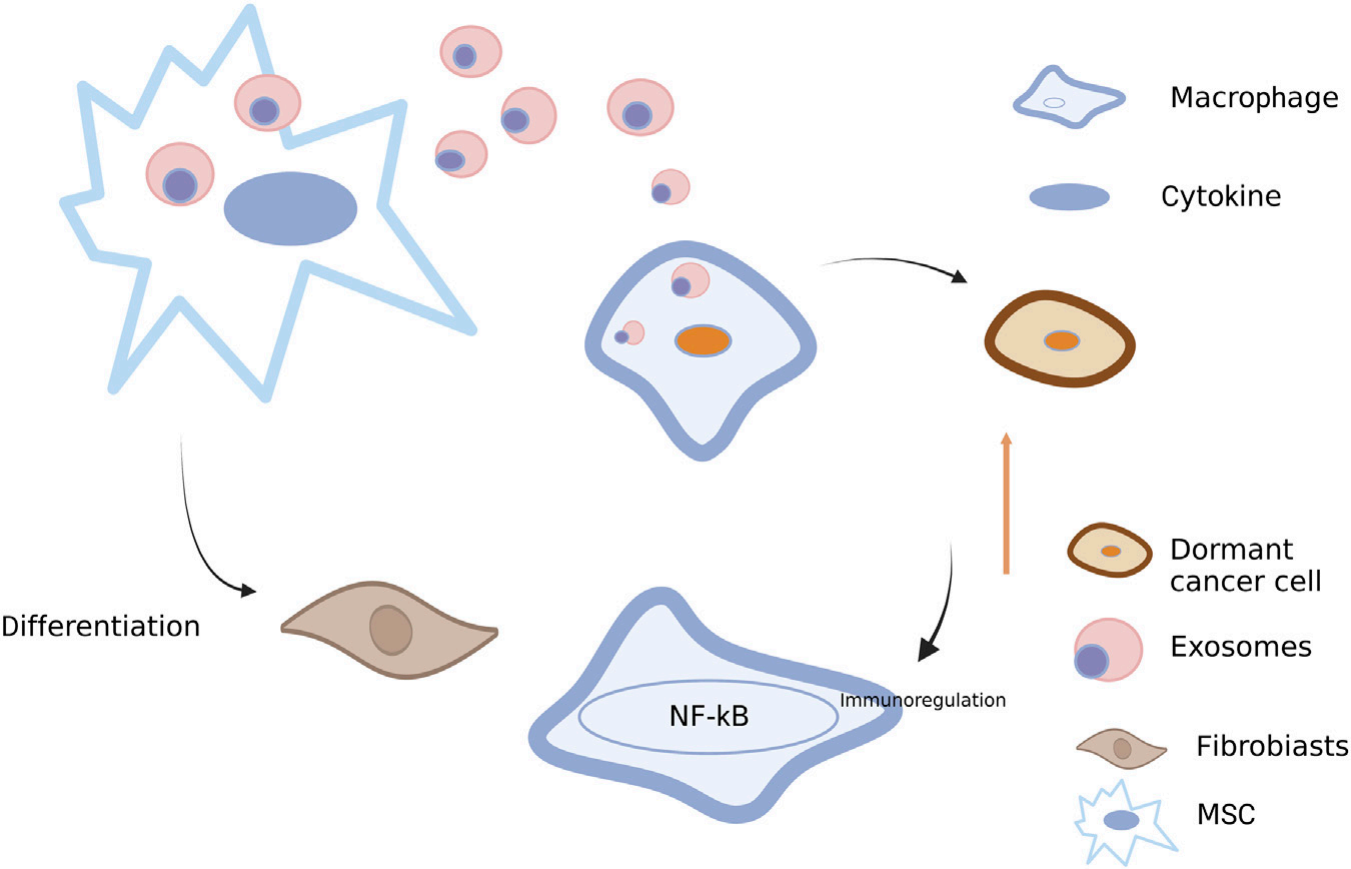
Roles of exosomes between BC cells and other stromal cells. Exosomes transferred from BC cells into macrophages may activate NF-κB, release cytokines and stimulate autoimmune regulation (Wang et al., 2018a). Exosomes from BC cells also play a role in mesenchymal stem cells (MSCs), thus promoting the differentiation into fibroblasts (Ochieng et al., 2009). Conversely, exosomes derived from MSCs can also act on BC cells, leading to the dormancy of BC cells (Ono et al., 2014). Created with BioRender.com.

As previously discussed, exosomes, which carry certain proteins or RNAs, mediate the induction of drug resistance. Restricting the release of exosomes from specific cell types may help mitigate the contribution of exosomes to the development of drug resistance in BC, such as BC and stromal cells (Datta et al., 2018; Sun et al., 2018), as well as by preventing the drug carrier’s integration into exosomes, which could lead to re-distribution and accumulation of the drug in BC cells (Kong et al., 2015; Koch et al., 2016) (Figure 5). The goal of this study was to assess the effects of exosome depletion on tumor cell proliferation, migration, and metastasis after treatment with platinum-based chemotherapy or combination therapy for advanced pancreatic adenocarcinoma (PAA). This method has been used successfully in patients with multiple sclerosis, and it was found to be effective in reducing the risk of relapse after chemotherapy or radiotherapy. In addition, their role in immunotherapeutic therapies, exosomes may also play an important role in cancer therapy. It is a secure and efficient natural carrier for therapy or targeted medication administration based on a particular exosome component. Li et al. (Li et al., 2020) created a poly (lactic-co-glycolic acid) nano platform coated with macrophage-derived exosomes for TNBC-targeted chemotherapy. Similar to this, To decrease the expression of miR-1423p and miR-150 in 4T1 and TUBO BC cell lines, Naseri et al. (Naseri et al., 2018) employed exosomes that were isolated from bone marrow-derived MSCs to transfer anti-miR-142-3p oligonucleotides that had been LNA (locked nucleic acid)-modified. Aqil et al. (Aqil et al., 2017) demonstrated that curcumin could be successfully delivered via milk-derived exosomes. In the present study which has shown that curcumin can also be delivered through exosome-derived extracellular vesicles (EVVs). These EVVs are a key part of cell membranes and play a significant part in cell functions, such as communication with other cells, differentiation of cells, regulation of gene expression, or migration. Oral delivery of exosomal curcumin showed superior anti-proliferative, anti-inflammatory, and anti-cancer activities against a variety of cancer cell lines including BC when compared to free curcumin. Exosomes can be loaded with a variety of peptides, non-coding RNAs, or chemotherapeutic medicines. They are an effective carrier to improve anticancer therapy and overcome drug resistance. In addition, their potential use in clinical trials, exosomes may also serve as new therapeutic targets for many other cancers and disorders such as BC, lung cancer, colon cancer, pancreatic cancer, prostate cancer, melanoma, and others (Zhong et al., 2016).

The transfer of tumor-associated antigens and major histocompatibility complex class I molecules to DC via tumor-derived exosomes has been demonstrated to activate the T cell-mediated immune system against tumor cells (Wolfers et al., 2001). Several researchers (Li et al., 2018) presented an alternative therapy option for BC patients who are resistant to trastuzumab and have a HER2-specific autoimmune tolerance.

### 6.3 Exosomes provide a new method for treating BC

Exosomes are effective therapeutic carriers due to their nanoscale size, immune compatibility, low toxicity, and relative durability. Exosomes from various cells can be loaded with a variety of non-coding RNAs, peptides, or chemotherapy medicines using a variety of techniques. To achieve the goal of treatment, researchers have developed the use of macrophage-derived exosomes as a drug delivery platform to transport platinum nanoparticles for specific use in BC and lung metastatic tumor cells, while activating cell apoptosis and inhibiting cell proliferation to inhibit the metastasis of BC (Xiong et al., 2019). Yang et al. (Yang et al., 2021) found that exosomes derived from CAR-T cells could effectively target mesothelial positive TNBC cells by secreting perforin and granzyme B, and the research data showed good killing efficiency and safety. Milano et al. (Milano et al., 2020) showed that ADM/trastuzumab encapsulated in mesenchymal progenitor cell-derived exosomes could significantly improve its cardiotoxicity and enhance its cytotoxic effect on BC cells in a rat model. Researchers have also discovered that PD-1 produced as exosomes in TNBC could prevent PD-L1-induced anti-tumor immunological dysfunction and increase the cytotoxic efficacy of effector T cells against tumor cells (Qiu et al., 2021). Therefore, More research is needed on the origin, production, and biological purpose of exosomes to support their clinical translation and application. Nanomaterials have a variety of roles in treatment, not only in BC but also in other cancers. For instance, Liu et al. (Liu et al., 2023a) developed a hybrid exosome-coated nanoplatforms based on zinc sulfide for the targeted treatment of *in situ* mouse glioblastoma models, demonstrating that HCQ @ ZnS @ eRGD stands out as a potent and all-encompassing therapeutic compound. A new therapeutic avenue for the treatment of glioblastoma is made possible by HCQ @ ZnS @ eRGD.

**FIGURE 6:**
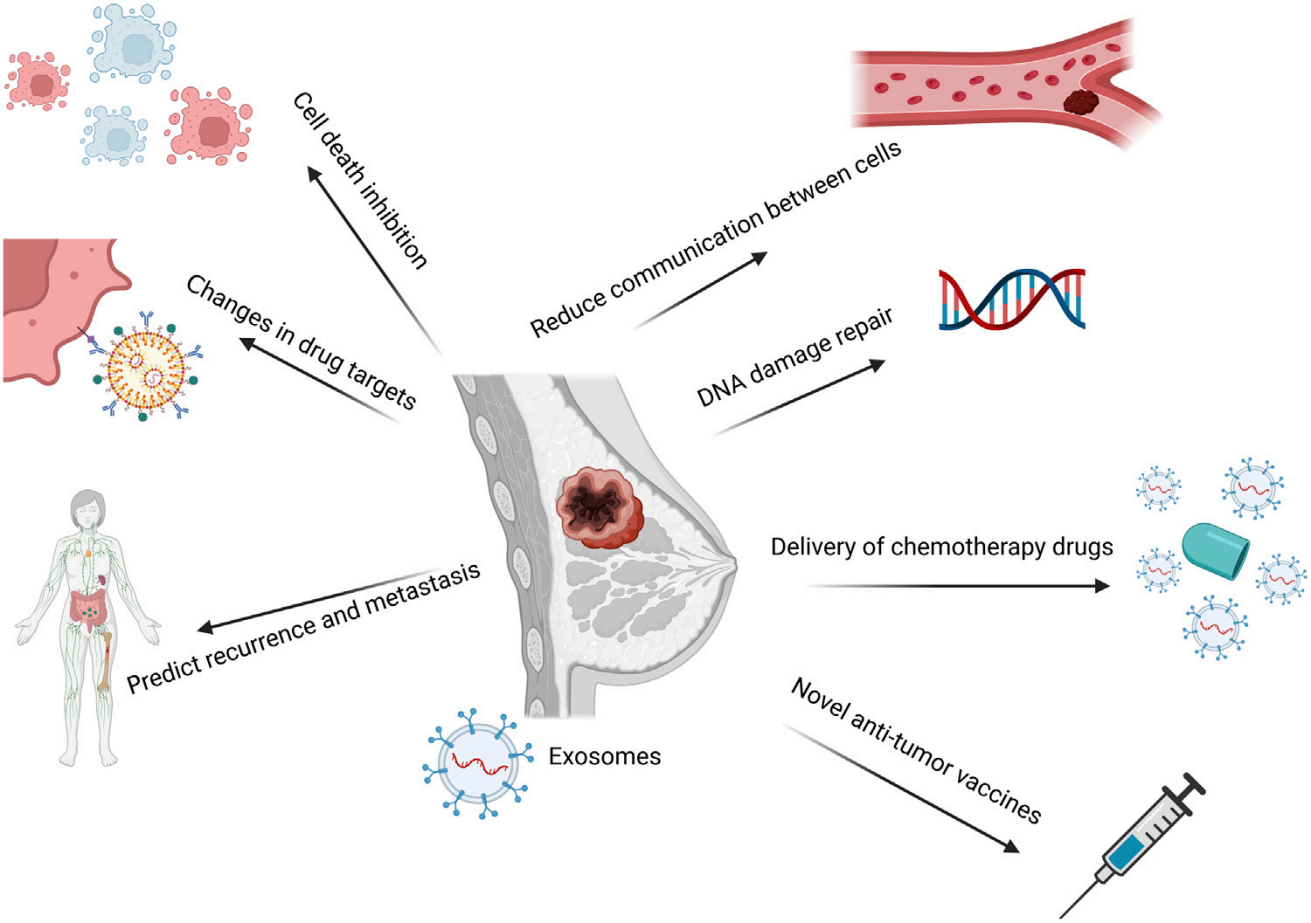
A summary of the exosome-mediated mechanism-based prospective treatment approaches for BC medication resistance. Exosomes-mediated drug delivery; finding new drug targets; altering the tumor microenvironment; keeping track of the recurrence and metastasis of BC cells; taking part in the epithelial-mesenchymal transition process; and other potential therapeutic strategies are based on the exosomes-mediated mechanism. Created with BioRender.com.

Based on the discussion above, future researches could be conducted in the treatment of BC drug resistance from multiple angles, primarily including: using exosomes as a drug delivery system; finding new drug targets; altering the tumor microenvironment; monitoring the recurrence and metastasis of BC cells; and taking part in the epithelial-mesenchymal transition. Exosomes as the transport medium could prevent the excessive loss of medications and obtain the maximum tumor cure rate by altering the conventional method of drug delivery and transportation. Additionally, it can play a role in DNA repair. Combining chemotherapy with the suppression of repair mechanisms could make cancer cells more responsive to the treatment and enhance the therapeutic outcome. Secondly, it may alter the medication targets. Exosomes have unique physiological properties, thus more basic researches on protein-related targets in exosomes are needed, and more theoretical support is needed for the clinical development of new drugs. Monitoring the systemic recurrence and metastasis of BC cells, searching for specific biomarkers that can detect recurrence and metastasis in time, and achieve early treatment of BC resistance to a certain extent. Exosomes could alter the current tumor microenvironment and reduce tumor medication resistance, recurrence, and metastasis in certain circumstances. Exosomes have been reported as a regulator of the immune response of tumor cells and as a new kind of tumor vaccination (Liu et al., 2022). Based on the exosome-mediated mechanism, exosomes have a wide range of potential applications in the prevention and treatment of BC resistance. The potential treatment strategies of BC drug resistance are summarized in Figure 6.

**FIGURE 7:**
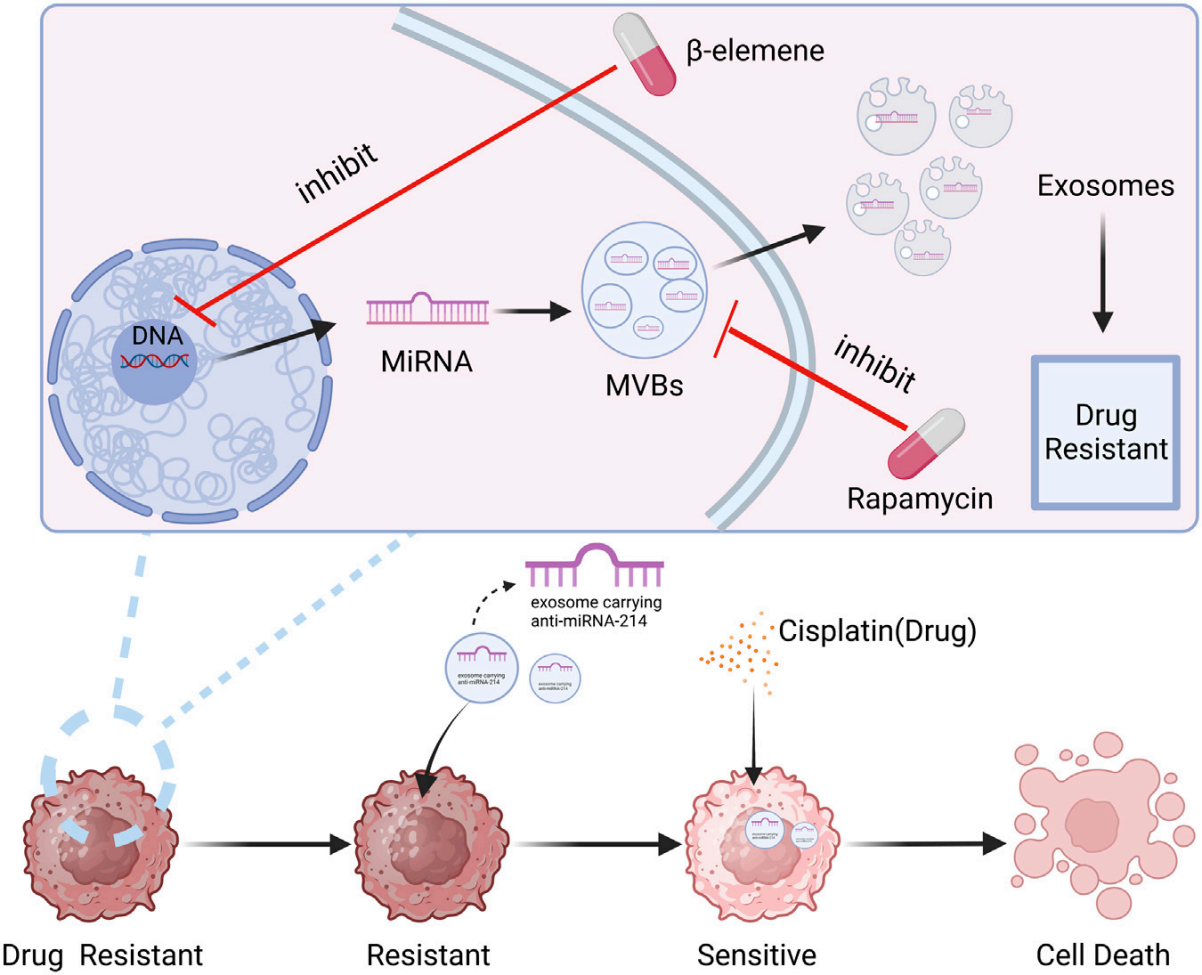
The role of exosomes in tumor drug resistance. It can be seen from the figure that exosomes, as a function of intercellular communication, can play an important role in anti-BC drug resistance. Created with BioRender.com.

Exosomes have proven to be potential nanocarriers that can be employed to reverse tumor treatment resistance, according to the research that is currently accessible. For example, Wang et al. (Wang et al., 2018b) sensitized cisplatin-resistant gastric cancer cells by directly delivering anti-miRNA-214 to the recipient cells through exosomes. Rapamycin and U18666A interfere with MVB production and cholesterol uptake into cell membranes, which can block exosome release and make B lymphoma cells more sensitive to rituximab. Researchers have discovered that b-element can modify the production of resistance-related miRNAs in exosomes by acting on certain genes in BC cell lines. This reduces the amount of resistance transmission through exosomes and increases the sensitivity to chemotherapy (Zhang et al., 2015). The role of exosomes in tumor drug resistance, has simply summarized in Figure 7.

## 7 Challenges and future perspectives

Exosomes is a “double-edged sword” in the treatment of BC drug resistance. The most common characteristics of malignant tumors are invasion and metastasis. Invasion and metastasis are multi-step processes that involve “crosstalk” between tumor cells and normal cells around the tumor at each stage. Exosomes, as one of the intercellular communication carriers, can directly transfer messenger RNA, miRNA, and proteins into cells and activate related signaling pathways, boosting tumor invasion and metastasis. To summarize, on the one hand, exosomes can induce drug resistance in BC; on the other hand, exosomes constitute a significant breakthrough in the treatment of BC drug resistance.

Exosomes have many application scenarios in the treatment of BC drug resistance. Firstly, exosomes are a type of EV that exist in the circulation system. Exosomes exist in all biological fluids and are secreted by all cells. They could be applied to the dynamic measurement of a variety of biological components related to tumor drug resistance, and have the unique potential to monitor the dynamic complexity of cancer. The biogenesis of exosomes can capture complex extracellular and intracellular molecules and can be used for comprehensive, multiparametric diagnostic assays. The surface proteins of exosomes also contribute to their immune capture and enrichment. Secondly, exosomes also have the potential to serve as candidate biomarkers for predicting and monitoring treatment effects in BC patients. Thirdly, the property of exosomes to deliver functional substances to diseased cells facilitates their use as therapeutic vehicles and as potential targets or transporters for reversing drug resistance. As a drug carrier, liposomes are a new type of targeted preparation that has been clinically applied earlier and is the most mature. Compared with liposomes, exosomes have a lower immune clearance rate. In addition, exosomes have been proven to be well tolerated and have no obvious side effects, opening up a new way to treat BC. Currently, exosomes have good prospects in treating BC drug resistance and treating drug resistance in other tumors, but there are also some difficulties. First, although there are many sources of exosomes, traditional extraction methods are insufficient to identify specific exosomes (Akagi et al., 2015). In this regard, it is necessary to find efficient, fast, and economical methods to clarify the source of exosomes, and more clinical studies are needed to verify the effectiveness and safety of current strategies for exosomes to deal with drug resistance in BC. At present, there are few clinical research reports on BC exosomes. In recent years, people have paid more and more attention to the function of exosome genes, but the relevant mechanisms have not been fully elucidated. Since conventional drugs are unavailable in many cases or patients develop an immune response to tumor cells after surgery, molecular targeted therapy may be an effective option for unstable cancer cells. Molecular targeted therapy can achieve cure by inducing the body’s own production of anti-exosome antibodies. However, there is no complete definition yet because this technology has some limitations: first, the method requires a long time and a lot of effort and high cost; secondly, exosomes may enhance the side effects of drugs and tumor cells of drug resistance. In addition, in most cases, exosomes must be controlled by specific gene expression products to function. However, currently, there are no systematic reports on the expression and metastasis of exocrine hormone receptor-binding proteins in tumors.

In summary, the study of exosomes is an active area of research and additional studies in the future may yield valuable information on their heterogeneity and biological functions and enhance the ability to exploit their therapeutic and diagnostic potential, Provide more ways and ideas for clinical treatment of BC drug resistance and other research fields. I believe that soon, it will bring good news to many cancer patients.
